# Nanoscale X-ray Imaging of Composition and Ferroelastic Domains in Heterostructured Perovskite Nanowires: Implications for Optoelectronic Devices

**DOI:** 10.1021/acsanm.3c02978

**Published:** 2023-09-21

**Authors:** Susanna Hammarberg, Lucas Atila Bernardes Marçal, Nils Lamers, Zhaojun Zhang, Huaiyu Chen, Alexander Björling, Jesper Wallentin

**Affiliations:** † Synchrotron Radiation Research and NanoLund, Lund University, Box 118, Lund 22100, Sweden; ‡ MAX IV Laboratory, Lund University, Lund 22100, Sweden

**Keywords:** perovskites, MHP, CsPbBr_3_, nanowires, heterostructures, scanning XRD, XRF

## Abstract

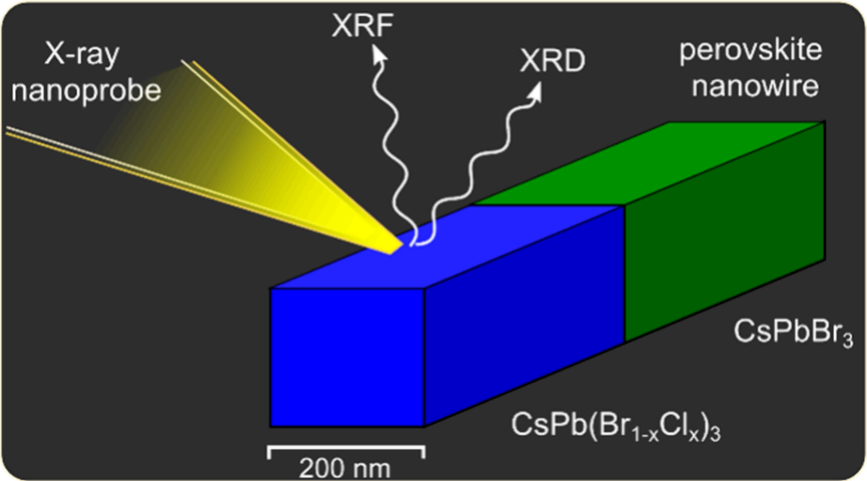

Metal halide perovskites (MHPs) have garnered significant interest as promising candidates for nanoscale optoelectronic applications due to their excellent optical properties. Axially heterostructured CsPbBr_3_–CsPb(Br_(1–*x*)_Cl_*x*_)_3_ nanowires can be produced by localized anion exchange of pregrown CsPbBr_3_ nanowires. However, characterizing such heterostructures with sufficient strain and real space resolution is challenging. Here, we use nanofocused scanning X-ray diffraction (XRD) and X-ray fluorescence (XRF) with a 60 nm beam to investigate a heterostructured MHP nanowire as well as a reference CsPbBr_3_ nanowire. The nano-XRD approach gives spatially resolved maps of composition, lattice spacing, and lattice tilt. Both the reference and exchanged nanowire show signs of diverse types of ferroelastic domains, as revealed by the tilt maps. The chlorinated segment shows an average Cl composition of *x* = 66 and *x* = 70% as measured by XRD and XRF, respectively. The XRD measurements give a much more consistent result than the XRF ones. These findings are consistent with photoluminescence measurements, showing *x* = 73%. The nominally unexchanged segment also has a small concentration of Cl, as observed with all three methods, which we attribute to diffusion after processing. These results highlight the need to prevent such unwanted processes in order to fabricate optoelectronic devices based on MHP heterostructures.

## Introduction

**Figure 1:**
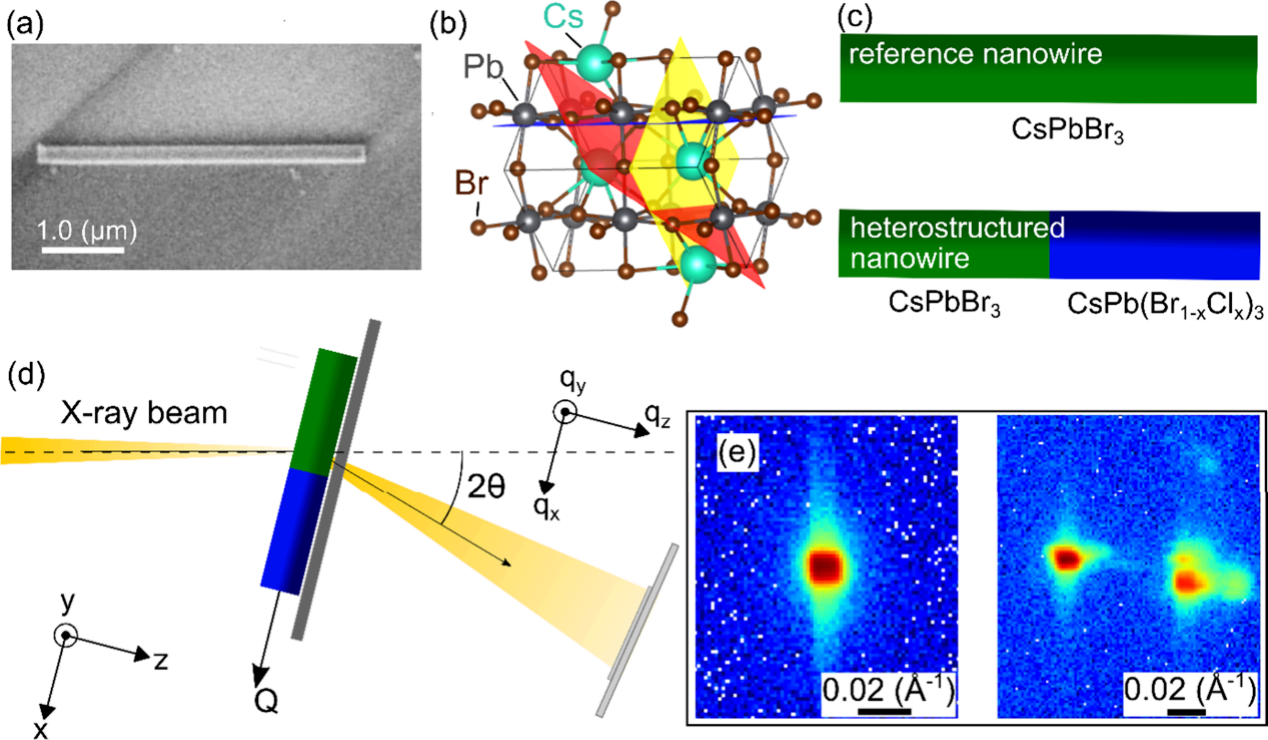
(a) Scanning electron microscope (SEM) image of a heterostructured nanowire of the same sample as the one investigated with nano-XRD and XRF. The exposure was kept short to minimize electron beam damage. (b) Sketch of orthorhombic unit cell of CsPbBr_3_ created in VESTA.^30^ (c) Sketch of a reference CsPbBr_3_ nanowire and a CsPbBr_3_–CsPb(Br_(1–*x*)_Cl_*x*_)_3_ nanowire heterostructure. (d) Scanning nano-XRD experimental setup. (e) Example of diffraction frames from the central part of the reference nanowire (left) and the heterostructured nanowire (right).

In the recent past, metal halide perovskites (MHPs) have become the topic of an avalanche of research into various optoelectronic applications. Most famously, MHP solar cells have reached high power conversion efficiencies (PCEs),^1^ with major advances in stability in recent years.^2^ The prominent PCE has been attributed to long charge carrier lifetimes and long diffusion lengths of charge carriers. MHPs are also investigated for other applications. CsPbBr_3_ is an MHP with a band gap of about 2.3 eV with possible applications in light-emitting diodes^3^ and X-ray scintillators.^4^ Growing MHPs in the shape of nanowires offers certain advantages compared with thin films, such as the guiding of light that can support single nanowire lasing.^5^ One method to grow nanowires is to use anodized aluminum oxide (AAO) templates,^6^ which can also lead to a substantial improvement in stability.^7,8^ Recently, we discovered that free-standing CsPbBr_3_ nanowires can grow from AAO templates, as shown in Figure 1a, offering a wide range of possibilities for device integration.^9^

Heterostructures are frequently used in traditional semiconductor devices to control the band structure, and they are essential for efficient optoelectronic devices, such as light-emitting diodes and lasers, by increasing radiative recombination. In traditional semiconductors, epitaxial heterostructures are typically created during the crystal growth. In MHPs, the composition of the soft ionic crystals can instead be modified after crystal growth using ion exchange,^10^ which can be combined with lithographic techniques to create nanowire heterostructures.^11^ The exchange is thought to proceed via vacancy-assisted diffusion mechanisms, which can lead to a blurring of the heterojunction^12^ but also to the formation of a core–shell structure.^13^ The processes of anion exchange and anion migration are not well understood. For instance, the heterostructures have inherent strain from lattice mismatch, which could affect the exchange process. An important question is how sharp heterojunctions can be formed since diffusion can proceed in undesirable directions. The heterojunction sharpness will also set a limit on how small segments can be created.

Another important difference between MHPs and traditional semiconductors is the lower symmetry of the crystal structure. The CsPbBr_3_ nanowires studied here have an orthorhombic crystal structure at room temperature, as shown in Figure 1b (*Pbmn*, *a* = 8.207, *b* = 8.255, and *c* = 11.759 Å). CsPbBr_3_ has two structural phase transitions, to tetragonal at 88 °C and to cubic at 130 °C. The low symmetry leads to ferroelasticity, i.e., the formation of crystal domains with different crystal orientations or phases that can be modified by external forces like mechanical pressure and a change in temperature or strain.^14^ Ferroelasticity has been shown to exist in MHPs,^15^ and recent studies suggest that this could suppress charge carrier recombination in solar cells.^16^ It is unclear whether the ferroelasticity affects the anion exchange or vice versa.

X-ray diffraction (XRD) is the method of choice to study both heterostructures and ferroelasticity due to its high reciprocal space resolution, but it has previously been plagued by poor real space resolution. However, with the brilliance of the fourth-generation synchrotrons, together with modern X-ray focusing capabilities reaching below 100 nm, variations of strain and lattice tilts can be imaged in nanostructures.^17−19^ Scanning nano-XRD is a technique to probe lattice spacings, strain, and lattice tilts in samples on the nanoscale with high resolution and superior strain sensitivity,^20−24^ and it has been used to image MHP nanowires to probe ferroelastic domains and phase transitions.^25−27^ With simultaneous scanning X-ray fluorescence (XRF) measurements, variations in a crystal can be correlated to compositional analysis.

Here, we investigate axial CsPbBr_3_–CsPb(Br_(1–*x*)_Cl_*x*_)_3_ nanowire heterostructures, which were recently demonstrated with an improved fabrication approach,^28^ using scanning nano-XRD and XRF with a 60 nm hard X-ray beam. Ferroelastic domains are observed in both the reference and the heterostructured nanowire. We observe that the heterostructure with two well-defined Bragg peaks is formed with a sharp and straight interface. The compositions, as measured by XRD, XRF, and photoluminescence (PL), show good agreement, albeit with significantly lower variation using XRD than with XRF, and we find that the nominally unchlorinated segment has a small unintentional Cl concentration.

## Experimental Section

First, free-standing CsPbBr_3_ nanowires were grown as previously described.^9^ An AAO membrane with 200 nm diameter pores was placed on top of a liquid precursor solution of CsBr and PbBr_2_ in dimethyl sulfoxide (DMSO) and then heated until CsPbBr_3_ nucleated inside the pores. Eventually, free-standing nanowires with lengths of 1–10 μm grow out of the nanopores. Previous laboratory source XRD has shown that the free-standing nanowires are single crystals and grow predominantly in the (001) direction.^9^ The nanowires have approximately square cross sections with {110}-type facets. The nanowires are removed from their substrate by scraping the surface with a tissue and transferring them to a fresh Si_3_N_4_ substrate. The nanowires were processed to form heterostructures in an anion exchange process as previously reported.^28^ In short, the samples were covered with a polymer resist, which was selectively opened in half of the nanowires using electron beam lithography in a process based on nonpolar solvents.^29^ Next, the samples were exposed to Cl_2_ gas with a 3.33 × 10^–5^ bar partial pressure at room temperature for 360 s, as previously described in detail,^28^ creating CsPbBr_3_–CsPb(Br_(1–*x*)_Cl_*x*_)_3_ heterostructures as sketched in Figure 1c.

The nanowires were characterized with the hard X-ray nanoprobe NanoMAX^31,32^ at the fourth-generation synchrotron source MAX IV Laboratory in Lund, Sweden. A schematic sketch of the experimental setup is shown in Figure 1d. An X-ray beam with a photon energy of 15 keV was focused using a Kirkpatrick–Baez (KB) mirror setup to 60 nm × 60 nm^2^. The beam focus was characterized with ptychography on a test sample which gave a reconstruction of the illumination with high resolution.^33^ The perovskite sample was mounted on a piezoelectric stage for lateral and rotational scanning and placed in the KB focus. Individual nanowires were oriented horizontally in the beam. The full incident flux was 1.24 × 10^10^ photons s^–1^, which we reduced with absorbers to 2.5 × 10^9^ photons s^–1^ to minimize beam damage on the nanowires. A Merlin Quad 2D pixel detector with 55 μm pixel size was placed on a robot arm in Bragg geometry at around 2θ = 17°, at a distance of 0.3000 m from the sample. The detector robot has an accuracy of 20 μm for absolute positioning precision and less than 10 μm for repeatability.^32,34^ At a detector distance of 300 mm, this should give an inaccuracy of about 10^–4^. Note that this does not affect the relative strain variations since the detector position was held fixed during the measurements. Simultaneously, XRF was measured with a detector close to the sample.

The 004 Bragg reflections of orthorhombic CsPbBr_3_ and CsPb(Br_(1–*x*)_Cl_*x*_)_3_ were measured to generate maps of the lattice spacing along the nanowire long axis. The geometry of our setup allowed us to simultaneously measure both Bragg peaks in the heterostructures (see an example in Figure 1e (right)). To measure the Bragg peaks in 3D, the sample was slightly rotated around the Bragg condition in small angular increments along the so-called rocking curve.

Data sets were measured from untreated reference CsPbBr_3_ nanowires and Cl_2_ exposed nanowires with CsPbB_3_–CsPb(Br_(1–*x*)_Cl_*x*_)_3_ heterostructures, and here, we present one nanowire of each type. For the scanning nano-XRD measurements, the sample was translated in the beam in a fly scanning mode in the *x*-direction, corresponding to a step size of 50 nm, and with a 50 nm step size in the *y*-direction, for the reference nanowire. The corresponding step sizes for the heterostructured nanowire were 60 nm × 60 nm. The exposure time at each scanning position was 0.01 s. The nanowires were rotated in increments of 0.1° in a range of around 2.0° for the reference nanowire and 2.5° for the heterostructure.

As a first step of the analysis, XRF maps from Pb and Br were used to align the diffraction to account for shifts in the sample position between rotations on the rocking curve. The strain and tilt maps were calculated from the Bragg peak positions in 3D reciprocal space for each lateral scanning position on the sample.^21,35^ The position of the Bragg peak for each point was found with a center of mass calculation, and maps of the lattice spacing and tilts were calculated. The lattice tilts α and β are defined as rotation around *q*_*z*_ and *q*_*y*_, respectively.

For PL measurements, a wide-field fluorescence microscopy setup was used. The nanowires were excited via a 40× NA0.6 objective lens using a defocused 405 nm CW diode laser with power density 3 mW/cm^2^ at the sample plane, and the same objective collected the PL spectra. The spot size of the excitation spot is around 10 μm, larger than the nanowires.

## Results and Discussion

An example of diffraction from the central region of the untreated reference nanowire is displayed in Figure 1e (left), where a single Bragg 004 peak is observed. The square-like shape of the Bragg peak originates from the slits before the KB mirrors. In contrast, the diffraction from the heterostructured nanowire in Figure 1e (right) shows two distinct 004 Bragg peaks, where the second Bragg peak at a higher *q*_*x*_ is due to the CsPb(Br_1–*x*_Cl_*x*_)_3_ segment. We investigated several reference and heterostructured nanowires, and in the following, we will discuss the results from one of each type.

### Reference Nanowire

**Figure 2:**
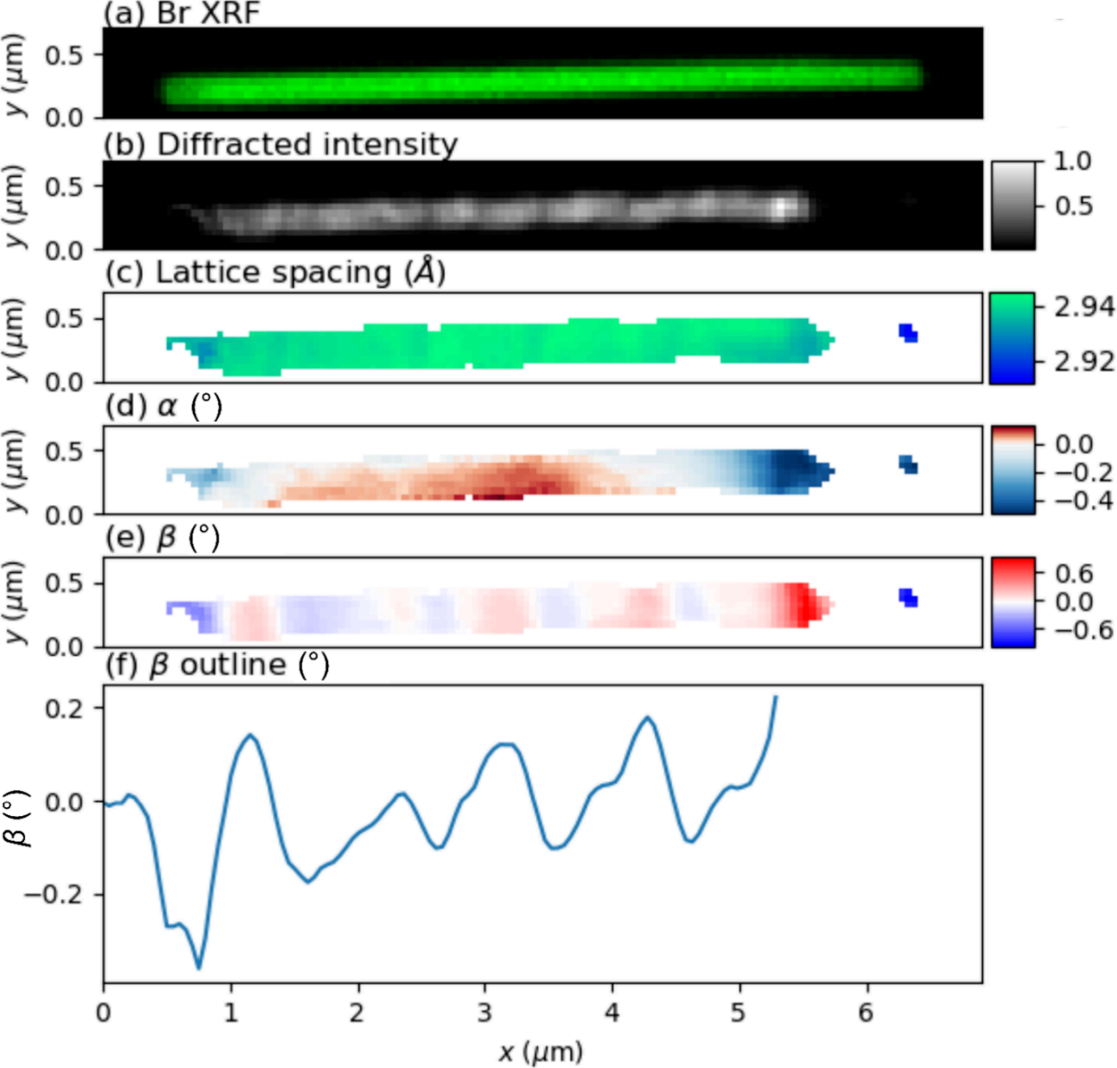
Scanning nano-XRD and XRF of a reference CsPbBr_3_ nanowire. (a) Normalized XRF signal from Br. (b) Normalized diffracted intensity. (c) Lattice spacing. (d) α lattice tilt, in the substrate plane. (e) β lattice tilt, perpendicular to the substrate. (f) β tilt along the nanowire long axis, averaged in the *y-*direction. The vertical range in this graph was optimized for the central part of the nanowire.

The reference CsPbBr_3_ nanowire is about 6 μm long and 200 nm in diameter (see Figure 2). The Br XRF map (see Figure 2a) displays a homogeneous composition, as expected. The integrated diffracted intensity of the Bragg peak for all angles (see Figure 2b) also shows a rather homogeneous distribution but with a gap at around *x* = 6.0 μm, which is not visible in the XRF map. As the XRF map is complete, there is no gap in the nanowire; rather, the lattice tilt in this region was so large that the crystal was out of the rocking curve range. The lattice spacing (see Figure 2c) is uniform throughout almost the whole nanowire, with an average value of *d* = 2.941 Å with a variation from +0.004 to −0.029 Å. The nanowire’s ends have slightly lower lattice spacing; around −0.3% in the left end and around −1% in the right end.

In contrast to the homogeneous lattice spacing, the lattice tilt β shows a systematic variation, as shown in the map in Figure 2e and more clearly in the vertical average in Figure 2f. In most of the wire, β repeatedly switches from around −0.10° to +0.15° along the nanowire long axis. Note also that small plateaus of constant tilt are observed between the peaks, e.g., at *x* = 4 μm. In III–V nanowires, which lack ferroic domains, morphological bending has been observed as rather long-range gradients in tilt.^36^ The long-range gradient in the tilt α that can be observed in Figure 2d is probably due to a slight in-plane bending. However, we assign the multiple sharp switches in lattice tilt in our MHP nanowires to ferroelastic domains, which are fundamentally related to tilting of octahedra.

From the above analysis, we observe that the pristine reference nanowire has a homogeneous lattice spacing profile with small variations, except for the ends, where the lattice spacing is slightly lower. The average value of *d* = 2.941 Å is very close to the expected 2.940 Å. This is in line with previous findings on similar CsPbBr_3_ nanowires.^27^ The deviating appearance and lower lattice spacing of the left end may indicate a distorted crystal from when the nanowire was broken off of the AAO template.

The systematic variation of the lattice tilt is a clear indication of ferroelastic domains. Here, we can distinguish between two types of domains. The right tip at around *x* = 4 μm has a different lattice spacing than the rest of the nanowire, with a value that conforms well with a 220 reflection. This suggests that the right top is a 110-oriented domain, which has previously been observed in CsPbBr_3_ nanoparticles.^37^ In the middle part of the nanowire, however, we observe systematic β variations without variation in lattice spacing, showing that the domains are separated with a relative lattice tilt but no change in the lattice constant. The constant lattice spacing indicates that all these domains present (001) *c*-planes orthogonal to the long axis. Note that the nanowires have previously been shown to have {110}-type facets,^9^ which will be parallel to the substrate surface. Therefore, adjacent domains could be rotated around the long axis in multiples of 90°, switching between different {110}-type facets. In this scenario, subsequent domains along the nanowire long axis will match, for instance, (110) with (−110), which could give rise to a tilt which can be calculated by^38^
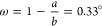
1

This value is comparable to the peak to valley β variation observed in our nanowire. The length scale of ferroelastic domains in CsPbBr_3_ nanowires has previously been shown to be on the order of 100–1000 nm.^25^ This is in line with the results here, with 12 domains in five microns, i.e., about 400 nm long domains, and widths across the nanowire diameter.

Regarding the domain wall orientation, previous investigations have shown that {112}-type domains are formed in CsPbBr_3_ nanostructures.^27,37,39^ In our geometry, such a domain wall would be oriented about 45° relative to the nanowire’s long axis and to the beam. Thus, when the beam illuminates a domain wall, both types of domains would contribute, and the average tilt is small. That would result in two tilts of opposite signs and a plateau between them, in agreement with our findings. However, we cannot exclude other domain wall orientations based on these results.

### Heterostructured Nanowire

**Figure 3:**
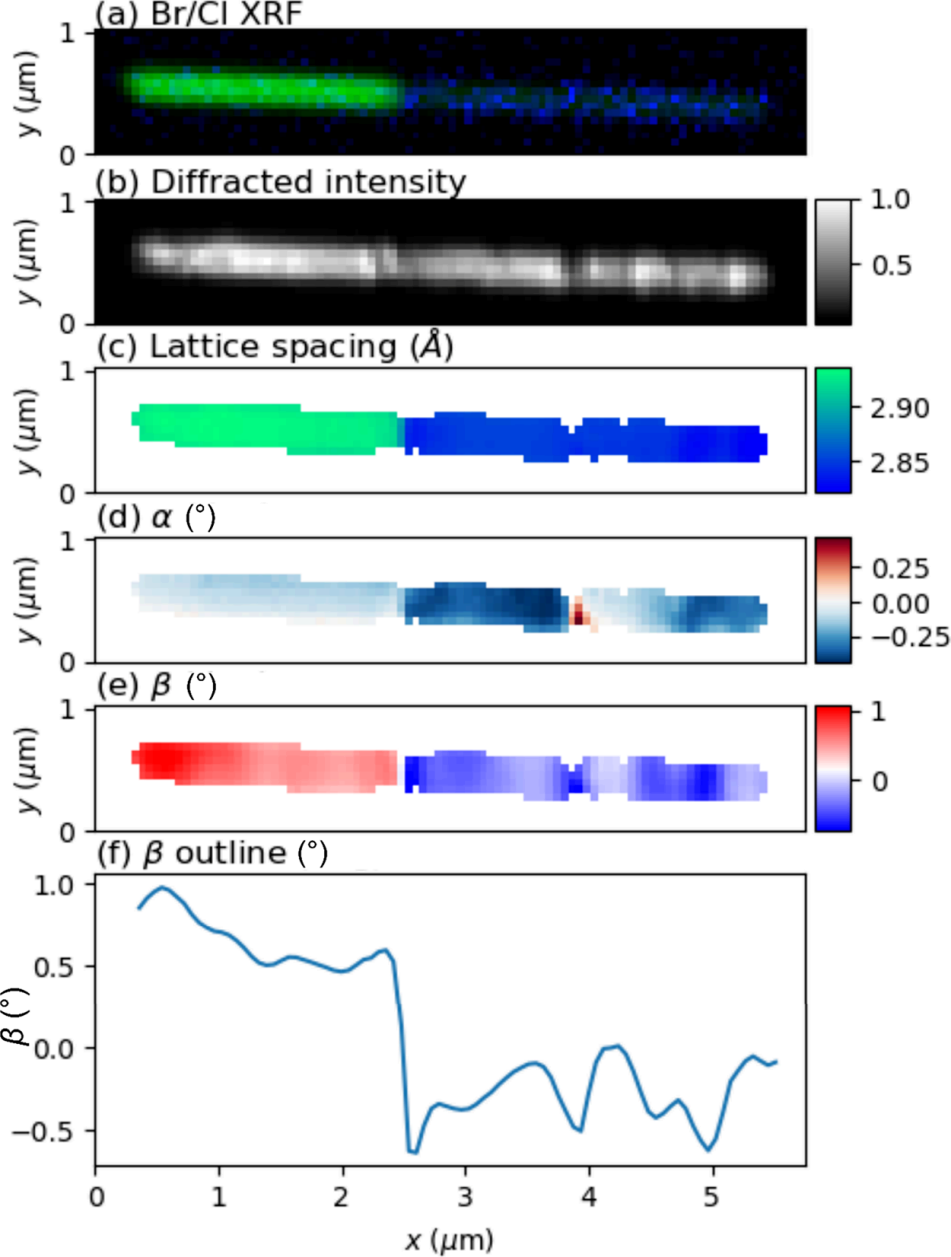
Scanning nano-XRD and XRF of the nanowire heterostructure. (a) Normalized XRF maps from Br (green) and Cl (blue). (b) Normalized diffracted intensity. (c) Lattice spacing. (d) α lattice tilt, in the substrate plane. (e) β lattice tilt, perpendicular to the substrate. (f) β tilt averaged in the *y* direction.

**Figure 4:**
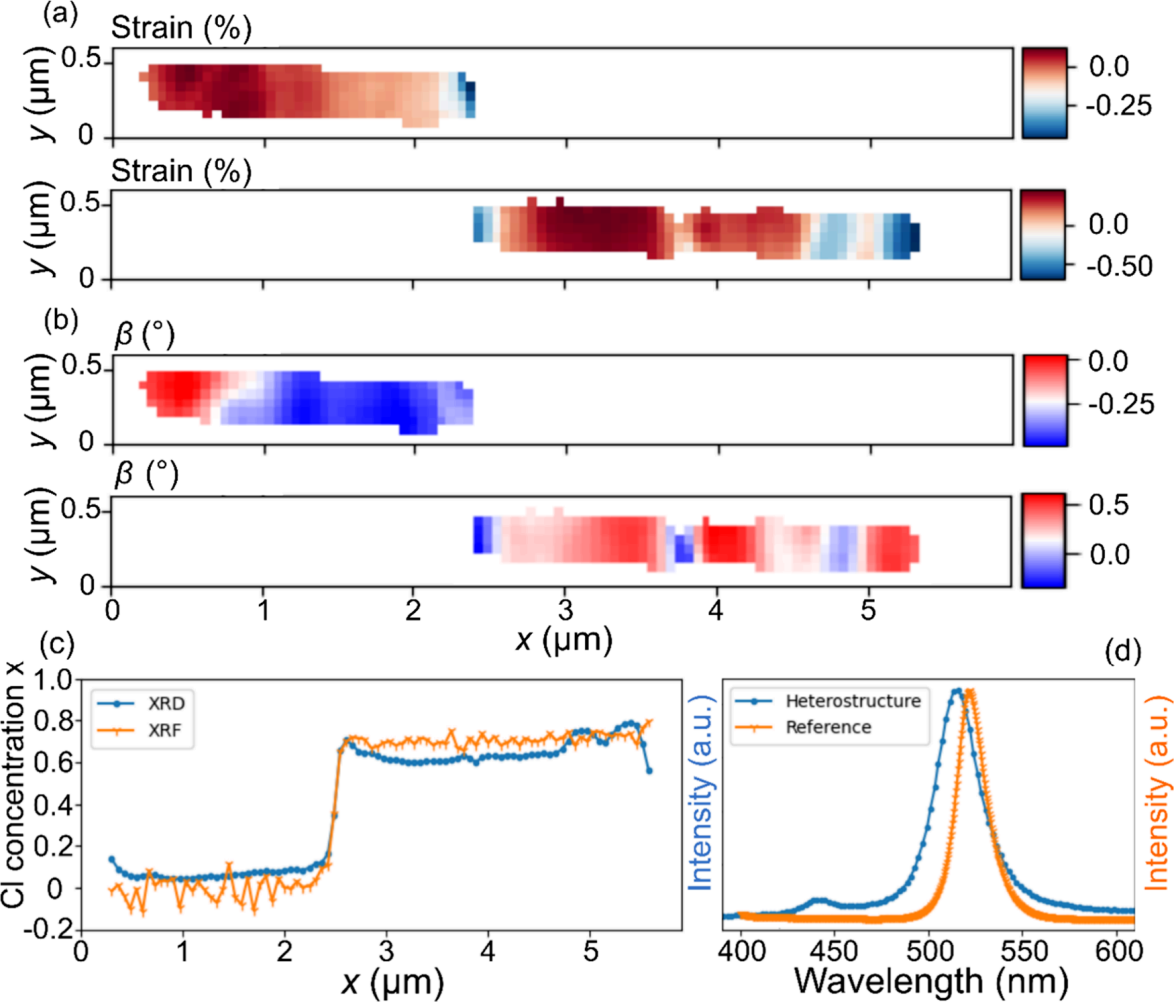
(a) Strain maps from the unchlorinated (top) and chlorinated (bottom) segment. (b) β tilt maps from the unchlorinated (top) and chlorinated (bottom) segment. (c) Concentration of Cl measured by scanning nano-XRD and XRF averaged in the *y-*direction. The XRF data are calculated from the Br to Pb ratio in comparison with the same ratio in the reference nanowire. (d) Photoluminescence from the reference and heterostructured nanowire, respectively.

The heterostructured nanowire is around 5.0 μm long and 200 nm in diameter. We present the results from XRF mapping and scanning nano-XRD in Figures 3 and 4. The XRF maps, as shown in Figure 3a, which have been summated from all rotations, show that the Cl (blue) has partially replaced the Br (green) in the right segment, creating a heterostructure with a sharp and straight interface around *x* = 2.5 μm. A minor concentration of Cl is also visible in the left, nominally unchlorinated, segment. The average lattice spacings (see Figure 3c) are *d* = 2.928 and *d* = 2.845 Å, in the unchlorinated and chlorinated segments, with variations of +0.008 to −0.023 Å and +0.014 to −0.022 Å, respectively. Note that the unchlorinated segment has a slightly lower lattice spacing compared with the reference nanowire.

The two segments show a significant difference in lattice tilt (see Figure 3d,e), with a split of about 1.2° in β and a smaller split of 0.2° in α. At around *x* = 4 μm, the α map unexpectedly deviates with slightly more positive values compared to the other homogeneous map (also visible in the example of the diffraction frame in Figure 1e (right), about 30 pixels above the right Bragg peak). The chlorinated segment shows a periodic variation in β, while the unchlorinated side is more homogeneous but with an axial gradient across the segment, as displayed more clearly in Figure 3f.

To better investigate strain and lattice tilt variations within the segments, scanning nano-XRD analysis was also performed on the two Bragg peaks separately, as shown in Figure 4a. Here, strain is calculated from the lattice spacing relative to the average in the respective segment and is affected by both the mechanical strain and the local composition. Both sides of the heterojunction show a compressive strain. The β maps from each Bragg peak are plotted separately in Figure 4b. The unchlorinated segment shows a single domain wall at about 500 nm from the nanowire tip, which in this case is diagonal. The chlorinated segment, however, shows multiple vertical and slightly diagonal lines, both with and without a change in lattice spacing.

The high resolution of the nanobeams makes it possible to evaluate the composition locally. We show the absolute Cl composition calculated from XRD and XRF in Figure 4c, both averaged over the nanowire in the *y-*direction for better statistics. The concentration from XRF was calculated from the Br to Pb XRF signal ratio in the nanowire compared to the reference nanowire. The relative concentration in the two measurements agrees reasonably well, although the XRF shows a large variation in the chlorinated segment. The Cl concentration from scanning nano-XRD was calculated with the assumption that the intermediate states between pure CsPbBr_3_ and pure CsPbCl_3_ are governed by Vegard’s law,^40^ using the average lattice spacing in the reference nanowire as a reference for pure CsPbBr_3_ and tabulated values for CsPbCl_3_ (orthorhombic *Pnmb, a* = 7.9136, *b* = 7.9145, and *c* = 11.1861 Å). From the XRD data, we calculate the average Cl concentration to *x* = 9 and *x* = 66% in the unchlorinated and chlorinated segments, respectively, while XRF gives average values of *x* = 3 and *x* = 70%, respectively. We find that the XRD shows a much lower variation than the XRF measurement.

As a third independent measurement of the composition, we used PL as shown in Figure 4d. The same nanowire was studied both using PL and at the beamtime. This is made possible by utilizing a marker system, which is deposited before any nanowires are transferred. The nanowires can then be uniquely identified by their position within this marker system, which is visible both in the PL setup and at the beamline. We measured the heterostructured nanowire in Figure 3, as well as a reference nanowire from the same growth as in Figure 2. The reference nanowire has green emission on average centered at 522 nm, similar to previous measurements.^9^ For the heterostructure, the unchlorinated segment has green emission on average that is centered slightly below the reference at 515 nm. The chlorinated side of the heterostructure has indigo emission, on average, centered at 440 nm. The Cl concentration was estimated to be *x* = 6% in the unchlorinated segment and *x* = 73% in the chlorinated segment, using Vegard’s law with the reference nanowire in Figure 4d and previously measured values for pure CsPbCl_3_ (410 nm^10^) as reference points. This is in reasonable agreement with the XRD and XRF results. Note also that the peak width for the unchlorinated segment is significantly larger, 28 nm, than that for the reference nanowire, 17 nm. In general, we do not observe any radial gradients in XRD or XRF, but it is difficult to draw any conclusions from this, as the beam size is about 30% of the diameter. The spatial resolution of micro-PL, about 500 nm, is not sufficient to reveal variations between the ferroelastic domains by XRD.

From the above analysis, we conclude that Cl has partially replaced the Br in the right segment, creating a heterostructure. This is manifested in the XRF map, the two separate Bragg peaks from XRD, and the two separated peaks in PL. The unchlorinated segment is concluded not to be pure CsPbBr_3_, as the average lattice spacing differs from the reference nanowire, and we observe a small amount of Cl in the unchlorinated segment in the XRF map. This is consistent with the offset between the green PL peak from the heterostructure and the reference peak. We conclude that a smaller fraction of the Cl has migrated to the unchlorinated segment, replacing a fraction of the Br anions there, most likely after the anion exchange processing of the nanowire. We propose that Cl has diffused from the chlorinated segment to the unchlorinated segment inside the nanowire, which is corroborated by the axial gradient of Cl that is observed in the unchlorinated segment in Figure 4c, in both the XRF and XRD signals, with a higher Cl level close to the interface. This is also consistent with a broadening of the green peak in the PL for the heterostructure compared to the reference peak. Similar observations have been reported at lower spatial resolution using micro-PL, where the diffusion was attributed to the soft ionic lattice of MHPs^41^ Since the processing was done at room temperature, it is unlikely that substantial diffusion occurred during this short time (360 s).

Both sides of the heterojunction show compressive strain. This is expected for the chlorinated segment as the lattice mismatch itself should lead to a tensile radial strain and compressive axial strain (which is the direction we probe here). However, for the nominally unchlorinated segment, this is unexpected. A possible explanation is a local diffusion of Cl that reduces the lattice distance, as discussed above. The observed lattice tilt at the heterojunction may also be induced by the lattice mismatch. Another possibility is the presence of a ferroelastic domain at the interface.

Not so many domains are visible in the unchlorinated segment (see Figure 4b), but the diagonal line indicates a (112)-type domain wall. On average, we retrieve a difference of about 0.3° from the subsequent domains around this domain wall, which is also in agreement with what is expected from eq 1 and the reference nanowire. The β map corresponding to the chlorinated segment, however, is much more complex. Although the spatial resolution does not allow for a more accurate analysis, a combination of both vertical and diagonal lines is seen along the nanowire. It is possible that they correspond to different types of (112) planes rotated around both the *y-* and *z-*axes. The tilt difference in subsequent domains is also very diverse in this segment.

## Conclusions

In conclusion, we demonstrated that nanofocusing scanning XRD and XRF can be used to image and quantify composition, strain, and ferroelastic domains within axially heterostructured MHP CsPbBr_3_–CsPb(Br_(1–*x*)_Cl_*x*_)_3_ nanowires. The demonstrated method is limited neither to nanowires nor to the CsPb(Br_(1–*x*)_Cl_*x*_)_3_ system and should be applicable to a wide range of MHP heterostructures and morphologies. Ferroelastic domains of different types were observed in both the heterostructure and reference nanowires. Unexpectedly, we observed that some Cl had diffused into the nominally unchlorinated segment. The compositions as measured by XRD, XRF, and PL show reasonable agreement for both segments, but XRD shows much less variation. PL has a lower spatial resolution. Also, the charge carriers will diffuse to the low-bandgap regions, especially in MHPs which have long diffusion lengths, making it challenging to use for measuring the local composition. The good agreement between PL and the composition as measured by XRD and XRF therefore implies that the composition is homogeneous on the nanoscale.

The heterojunction is sharp at the level of the 60 nm spatial resolution, although presumably diffusion leads to a certain blurring. The undesired diffusion of Cl is a challenge for optoelectronic devices based on MHP heterostructures since it reduces control of the bandgap. Furthermore, blurred heterojunctions could prevent the formation of short segments and quantum wells. New methods are needed to achieve sharper junctions and reduce undesirable diffusion, which could for instance be based on vacancy filling.^42^ Our results show that modern synchrotron methods can be used to guide the development of such processes.
